# LC-MS Identification and Quantification of Phenolic Compounds in Solid Residues from the Essential Oil Industry

**DOI:** 10.3390/antiox10122016

**Published:** 2021-12-19

**Authors:** Maria Irakli, Adriana Skendi, Elisavet Bouloumpasi, Paschalina Chatzopoulou, Costas G. Biliaderis

**Affiliations:** 1 Hellenic Agricultural Organization, Demeter, Plant Breeding and Genetic Resources Institute, P.O. Box 60458, Thermi, 57001 Thessaloniki, Greece; antrsken@teiemt.gr (A.S.); elisboul@abo.ihu.gr (E.B.); chatzopoulou@ipgrb.gr (P.C.); 2 Department of Food Science and Technology, International Hellenic University, P.O. Box 141, 57400 Thessaloniki, Greece; 3 Department of Food Science and Technology, Aristotle University of Thessaloniki, P.O. Box 235, 54124 Thessaloniki, Greece; biliader@agro.auth.gr

**Keywords:** phenolic acids, flavonoids, solid residues, essential oil industry, *Lamiaceae*, LC-MS, antioxidant activity

## Abstract

Plant solid residues obtained from the essential oil industry represent a rich source of phenolic compounds with bioactive properties to be used in the food and pharmaceutical industries. A selective and sensitive liquid chromatography-mass spectrometry (LC-MS) method was developed for the simultaneous determination of phenolic compounds in solid residues of the *Lamiaceae* family plants. A total of 48 compounds can be separated within 35 min by using the Poroshell-120 EC-C_18_ column, and a gradient mobile phase of 0.1% formic acid and acetonitrile with flow rate of 0.5 mL/min; salicylic acid was used as internal standard. The calibration curves showed good linearity in the tested concentration range for each analyte (R^2^ > 0.9921), while recoveries ranged from 70.1% to 115.0% with an intra-day and inter-day precision of less than 6.63% and 15.00%, respectively. Based on the retention behavior, as well as absorption and mass spectra, 17 phenolic acids, 19 flavonoids and 2 phenolic diterpenes were identified and quantified in the solid residues obtained by distillation of six aromatic plants: oregano, rosemary, sage, satureja, lemon balm, and spearmint. The method constitutes an accurate analytical and quality control tool for the simultaneous quantitation of phenolics present in solid waste residues from the essential oil industry.

## 1. Introduction

Aromatic plants of the *Lamiaceae* family are rich in phytochemicals, especially in phenolic compounds [1]. Moreover, phenolic compounds present in these plants are widely recognized for their high biological activity, such as antioxidant [1,2,3], anti-inflammatory, and antimicrobial as well as antifungal properties [3,4,5]. Additionally, the essential oils from plants of the *Lamiaceae* family, produced mainly by steam distillation, are rich in aromatic compounds with distinct flavoring and bioactive properties, for which there is an increasing demand nowadays in many industries (food and beverages, cosmetics, pharmaceuticals, perfumes, food and feed additives—supplements industry).

The production of essential oils from plants of the *Lamiaceae* family and especially from oregano (*Origanum vulgare* L.), rosemary (*Rosmarinus officinalis* L.), sage (*Salvia fruticosa Mill*), satureja (*Satureja thymbra* L.), lemon balm (*Melissa officinalis* L.), and spearmint (*Mentha spicata*) can be a profitable activity of the agricultural sector, contributing to the economic development in the Mediterranean region where these plants are well adopted and mainly grown. Nevertheless, the increased production of essential oils results in a great waste disposal of solid plant residues that remain after the extraction of essential oil, thus causing significant environmental concern. The extraction of phenolics from such waste represents an interesting and innovative valorization scheme as an opportunity to obtain high-added value products from these low-cost raw materials [6]. Until now, studies have dealt mainly with different extraction processes applied to waste residues, in order to obtain extracts with high antioxidant properties [7,8,9,10]. In these studies, the phenolic compounds present in the extracts are quantified in bulk utilizing mainly spectroscopic methods or antioxidant assays. Some of them involve quantification approaches using High-Performance Liquid Chromatography (HPLC) methods alone, or coupled with mass spectrometry (MS) or MS/MS. These methods aim mainly to identify the compounds present in the extracts. In the case, when quantification is performed, the external standard method is employed with the calibration curve for only a few main phenolic compounds supposedly present in the residues. Thus, these methods are not tailored for quantification of a great number of phenolics or even when developed for testing a great number of compounds, they are not validated to be applicable for routine analysis of this type of analytes remaining in the solid residue matrix, after the distillation of essential oils.

It is generally accepted that isolation and quantification of target analytes from different plant matrices require the development of appropriate methods that are characterized by high selectivity and sensitivity. Moreover, prior to the application of an optimized chromatographic method, appropriate extraction and purification techniques should be developed, in order to extract quantitatively all the analytes from the composite plant matrix.

The literature reports different extraction procedures for phenolics utilizing conventional liquid extraction involving organic solvents, such as methanol, ethanol, acetone, ethyl acetate, butanone, hexane as well as mixtures of them with or without water [11]. Conventional extraction of phenolics may be also coupled with Soxhlet extraction or without it [12,13,14] as well as with the application of new technologies such as microwave [15,16,17] or ultrasound [1,16,17], in order to reduce the time and energy required to obtain phenolic extracts. Moreover, supercritical fluid extraction (SFE) is used to extract phenolics utilizing CO_2_ as supercritical fluid alone or with ethanol as an entrainer to modify polarity and to enhance the yield of extracted phenolics [14,18,19,20,21].

Although a variety of techniques and analytical protocols are being applied in different laboratories for extraction, identification, and quantification of phenolics from individual aromatic plants, there is a need for a validated method to be used, according to internationally agreed standards. This method will not only be applied to identify individual compounds, but also to quantify a great number of phenolic constituents present in solid residues remaining after the essential oil distillation. On the other hand, a comprehensive profiling of the residual biomass that remains after the production of some important essential oils is still missing, due to their complex nature, the variability of cultivars or the chemical constitution of aromatic plants, the origin of plant material, etc., as well as the unavailability of commercial standards for proper identification and validation of these phenolic compounds. This work can contribute to the development of an alternative valorization scheme for these residual biomasses, in the content of circular economy to effectively address the problems of their disposal in an economically viable and environmentally sustainable manner. The potential exploitation of bioactive compounds, present in distillation residuals, by extraction of phytochemicals and further conversion into value added products, could find many applications in the food, pharmaceutical and cosmetic industries.

Thus, the main aim of the present study was to develop and validate an LC-MS method that will help to identify and quantify a total of 48 phenolic compounds recovered from solid residues after the extraction of essential oils of six plants of the *Lamiaceae* family. In this context, the objective was to establish and validate a simple method for analysis of phenolics at ng/mL levels and to apply this to real samples. The use of the MS detector permitted higher accuracy and confidence in the identification of analytes. Moreover, this study presents the application of the developed method for quantification of phenolic compounds in solid waste materials obtained from essential oil production of six different, commercially important aromatic plants of the *Lamiaceae* family (rosemary, sage, oregano, satureja, spearmint, and lemon balm). Since phenolics are linked with the antioxidant activity of plant extracts, the antioxidant potential of the obtained extracts was evaluated by three separate assays (DPPH, ABTS and FRAP) and related with the amount of phenolics determined by LC-MS and spectroscopic methods (Folin-Ciocalteu’s and aluminum complexation methods).

## 2. Materials and Methods

### 2.1. Chemicals and Reagents

Analytical standards of caffeic acid (CA), syringic acid (SRA), p-coumaric acid (pCA), ferulic acid (FA), sinapic acid (SA), 4-hydroxybenzoic acid (4HBA), salicylic acid (SLA), myricetin (MYR), quercetin (QUE), naringenin (NAR), chrysin (CRY), and verbascoside (VER) were obtained from Sigma-Aldrich (Steinheim, Germany). Quinic acid (QA), rosmarinic acid (RMA), neochlorogenic acid (nCLA), chlorogenic acid (CLA), cryptochlorogenic acid (cCLA), eriodictyol (ERY), apigenin-7-*O*-glucoside (APIGLU), galangin (GAL), rutin (RUT), apigenin (API), kaempferol (KAE), catechin (CAT), epicatechin (EPI), querce-tin-3-*O*-glucopyranoside (QUEGLU), hyperoside (HYP), epigallocatechin (EGCAT), and luteolin-7-*O*-glucoside (LUTGLU) were purchased from Extrasynthese (Genay Cedex, France).

Luteolin-7-*O*-rutinoside (LUTRUT), vicenin-2 (VIC), gallocatechin (GCAT), 1-caffeoylquinic acid (1-CQA), 3,5-dicaffeoylquinic acid (3,5-DCQA), 3,4-dicaffeoylquinic acid (3,4-DCQA), 4,5-dicaffeoylquinic acid (4,5-DCQA), isorhamnetin-3-rutinoside (ISRUT), isorhamnetin-3-*O*-d-glucoside (ISGLU), dihydrokaempferol (DHKAE), carnosol (CARO), and carnosic acid (CARA) were obtained from Carbosynth (Berkshire, United Kingdom). Vanillic acid (VA) was from Fluka (Buchs, Germany), naringin (NARI), and gentisic acid (GNA) were from Supelco (Bellefonte, PA, USA), whereas protocatechuic acid (PRCA), gallic acid (GA), and luteolin (LUT) were purchased from Alfa Aesar (Karlsruhe, German). Hesperidin (HESP) and trans-cinnamic acid (CNA) were obtained from TCI (Zwijndrecht, Belgium). Formic acid, methanol, acetonitrile and water of LC-MS grade were purchased from Sigma-Aldrich (Steinheim, Germany).

### 2.2. Standard Solutions

The standard stock solutions (1 mg/mL) of the phenolic compounds were prepared using methanol as a solvent and stored at −20 °C. Aliquots of each stock solution were mixed in order to prepare standard mixtures at concentration level of 100 μg/mL and stored at −20 °C. Mixed working solutions of phenolics were prepared freshly in methanol as a dilution series at approximate concentration of 0.01, 0.05, 0.1, 1, 2, 4, 5, 10 and 20 μg/mL or 25, 50, 75, 100, 200 μg/mL for carnosol and carnosic acid, and each contained 1 μg/mL of salicylic acid as internal standard (IS).

### 2.3. Plant Material

Aerial parts of six plant species belonging to the *Lamiaceae* family, i.e., Greek oregano (*Origanum vulgare* ssp. *Hirtum* L.), rosemary (*Rosmarinus officinalis* L.), sage (*Salvia fruticosa Mill*), satureja (*Satureja thymbra* L.), lemon balm (*Melissa officinalis* L.), and spearmint (*Mentha spicata*), were collected from cultivated accessions of Hellenic Agricultural Organization—Demeter, Institute of Plant Breeding and Genetic Resources (Thessaloniki, Greece). Based on the nature of the specific plants’ tissue, and the commonly applied essential oil distillation protocol, air-dried plant parts of oregano, rosemary, and sage, and fresh lemon balm and spearmint were subjected to steam distillation in a pilot scale essential oil distillation apparatus, i.e., ~2.5 Kg of each plant material was loaded in distillation still and the duration of distillation was approximately 2 h. The wet solid residue of each plant was collected after the steam distillation and was then sun-dried for 48 h. The dried material (~10% moisture content) was ground to pass through a 0.5 mm sieve in a laboratory mill (Retsch, Model ZM1000, Haan, Germany) and stored at 4 °C until further analysis.

### 2.4. Extraction Procedure

Samples of dried and ground solid residues samples (0.05 g) were extracted with 10 mL 70% methanol for 15 min at 30 °C using an ultrasonic bath (frequency 37 kHz, model FB 15051, Thermo Fisher Scientific Inc., Loughborough, England). The extract was then centrifuged at 10,000× *g* for 10 min at 4 °C and the extraction was repeated one more time. The clear supernatants were mixed, filtered through membrane filter with porosity of 0.45 µm, diluted with the standard solution of IS and either subjected directly to LC–MS analysis or stored at –20 °C until analysis.

### 2.5. LC-MS Analysis

Separation and detection of different phenolics present in the aforementioned extracts was performed by a Shimadzu Nexera HPLC system (Kyoto, Japan), which consists of two LC-30AD pumps, DGU-20A5 degasser, CTO-20AC column oven, SIL-30AC auto injector, SPD-M40 diode array detector (DAD) and a triple quadrupole mass spectrometer (model LCMS-2020), which was operated with an electrospray ionization (ESI) interface.

Each sample was eluted through a Poroshell 120 EC-C_18_ column (4.6 × 150 mm, 4 μm) with a column temperature of 35 °C and a flow rate of 0.5 mL/min. The injection volume was 10 μL and the mobile phase consisted of (solvent A) aqueous formic acid (0.1%, *v*/*v*) and (solvent B) acetonitrile. The adopted gradient program was as follows: 0–5 min, 15–25% B; 5–10 min, 25–35% B; 10–28 min, 35–60% B; 28–28.01 min, 60–15% B; and an isocratic elution until 35 min. The DAD acquisition ranged from 190 to 400 nm in steps of 1.2 nm. The mass spectrometer was equipped with an ESI source recorded on a negative ionization mode with +4.5 kV and 20 V interface and curved desolvation line (CDL) voltages, respectively. High-purity nitrogen (N_2_) was used as the nebulizing gas at a flow rate of 1.5 L/min and nitrogen (N_2_) was used as drying gas at a flow rate of 15 L/min. The block heater temperature and CDL temperature were maintained at 200 °C and 250 °C, respectively. Mass acquisitions were performed in full scan mode (100–1000 *m*/*z*) and selective ion monitoring mode (SIM). Data acquisition and processing was carried out using Lab Solutions LC-MS software (Shimadzu, Kyoto, Japan).

Samples were subjected to MS scanning for compound identification. The main phenolic compounds of samples were identified by comparing their retention time, UV profile and mass spectra of unknown peaks with those of authentic standards or with literature data. The majority of the phenolics detected were quantified using the calibration curves of corresponding standard solutions. When standards were unavailable, compounds with similar structure were used instead to perform quantification of the phenolic compounds. Specifically, lithospermic acid isomers, as well as sulphated RMA (rosmarinic acid), were quantified as RMA equivalents, whereas gallocatechin (GCAT) isomer and medioresinol were reported as GCAT equivalents.

### 2.6. Method Validation

The LC-MS method was validated in terms of linearity, limit of detection (LOD) and quantification (LOQ), accuracy, precision and recovery, according to the guidelines of the International Conference on Harmonization [22]. Linearity was assessed using internal standard calibration curves with six concentration levels for each analyte, and each concentration level was assayed in triplicate. Calibration curves were obtained by dividing the chromatographic peak area of the analyte by the corresponding peak area of the internal standard (1 μg/mL). Linear regression analysis was used to determine the slope, intercept and the correlation coefficient of each calibration line. The calculations for limit of detection (LOD) and limit of quantification (LOQ) were based on the standard deviation (SD) of y-intercepts of regression analysis (r) and the slope (S), using the equations LOD = 3.3 r/S, LOQ = 10 r/S, respectively, where r is SD of the intercept and S is the slope.

The precision and accuracy were determined by using three replicates of mixed standard solution at three levels on the same day (intra-day) and between 4 different days (inter-day) and finally assessed using the calibration curve. The precision of intra-day and inter-day were expressed as RSD (relative standard deviation) and accuracy was evaluated by comparing the calculated concentration with the standard concentration. The RSD values should not be more than 15% and accuracy should be in the range of 85% to 115%.

In addition, to evaluate the recovery values, known amounts of the analytes were added to the oregano, rosemary, and a mixture sample consisting of equivalent quantities of the six samples tested (oregano, rosemary, sage, lemon balm, satureja, and spearmint), using a mixed standard solution at high, medium and low concentration levels for each phenolic compound, according to their calibration curves. The recovery was calculated as the difference between the concentrations of analytes measured for spiked (Cs) and blank sample (Cb) divided by the theoretical spiked concentration (Ct) of the sample, and multiplied by 100, where Cs and Cb were calculated, according to the calibration curves [Recovery (%) = (Cs − Cb)/Ct × 100].

### 2.7. Determination of Total Phenolic and Flavonoid Content

Total phenolic contents (TPC) were analyzed with the Folin-Ciocalteu’s phenol reagent method, using gallic acid as the standard as reported in Skendi et al. [1]. The results were expressed as milligrams of gallic acid equivalents per g sample on a dry weight basis (mg GAE/g dw). The total flavonoid content (TFC) was determined by the aluminum complexation method as explained in Skendi et al. [23]. The results were expressed as milligrams of catechin equivalents per g of sample on a dry weight basis (mg CATE/g dw). Each determination was performed at least in triplicate.

### 2.8. Determination of Antioxidant Activity

The antioxidant activity of the extracts against 2,2-diphenyl-1-picrylhydrazyl (DPPH) radical, 2′-azino-bis-3-ethylbenzthiazoline-6-sulphonic acid (ABTS) reagent and FRAP (Ferric Reducing Antioxidant Power) solution was measured as described by Irakli et al. [24]. Antioxidant activity (DPPH, ABTS and FRAP tests) was expressed as mg Trolox ((S)-(-)-6-hydroxy-2,5,7,8-tetramethylchroman-2-carboxylic acid) equivalents per g of sample on a dry weight basis (mg TE/g dw). Measurements were performed at least in triplicate.

### 2.9. Statistical Analysis

The values presented in tables or figures refer to mean ± standard deviations of three parallel measurements. One-way analysis of variance (ANOVA) was used to test for differences among the means for different extracts, according to the Duncan’s multiple range test. Possible correlations between different groups of phenolics as measured with LC-MS and spectrophotometric methods and antioxidant activity were evaluated by Pearson’s and two-tailed significance coefficients. Data were tested using SPSS Statistics software version 19 (IBM SPSS Inc., Chicago, IL, USA); differences at *p* ≤ 0.05 were considered significant.

## 3. Results and Discussion

### 3.1. Method Validation Parameters

Extraction and purification of plant phytochemicals are considered crucial for the analysis of samples derived from complex matrices, especially when the aim is the simultaneous detection and quantification of multiple compounds. Factors such as ultrasound frequency, type of organic solvent, solvent concentration, extraction time, extraction temperature and solid-to-liquid ratio were considered in the present study in order to optimize the extraction procedure. The developed extraction procedure for LC-MS analysis of 48 phenolic compounds from the solid residue of the six aromatic plants after the essential oil distillation involved the use of ultrasonic extraction at 37 kHz with 70% aqueous methanol for 15 min and solid–liquid ratio of 1:250, as it was shown to be more efficient in analytes recoveries and thus was chosen throughout the experimentation.

To optimize the chromatographic analysis, the LC-MS conditions were initially established. First, two mobile phases consisting of acetonitrile, methanol and water, as acidified with various acids (formic acid and acetic acid), were tested. A combination of acetonitrile and 0.1% (*v*/*v*) aqueous formic acid was chosen, since acetonitrile gave better peak definition and resolutions. The amount of 0.1% (*v*/*v*) aqueous formic acid is also suggested by other authors [25] for compound detection in the negative ion mode, since it increases the ESI efficiency.

The developed LC-MS analytical method was efficient for the identification and quantification of 48 phenolic compounds. The method was validated according to International Conference on Harmonization (ICH) guidelines [22]. Thus, the linearity, the limit of detection (LOD), the limit of quantification (LOQ), intra- and inter-day precisions and accuracy, as well as the recovery were investigated.

Linearity represented by coefficients of correlation (R^2^) listed along with the calibration curve equations, the linear ranges, the LOD, and the LOQ are shown in Table 1. Within the investigated concentration ranges, all compounds showed good linearity with R^2^ values ranging between 0.9921 (VER) and 0.9999 (CLA and 3,4-DCQA). On the other hand, the LOD values ranged from 6.1 (ERY) to 718.3 ng/mL (CARA), while the LOQ values ranged from 18.5 to 2176.7 ng/mL for the studied 48 analytes. This fact reflects the high sensitivity of the developed method. Values of correlation coefficients were higher, whereas the LODs and LOQs observed in the present study were lower than those reported in another study, where 27 phenolic compounds were taken into consideration for LC-MS/MS detection and quantification in methanolic extracts of three *Salvia* L. species [26]. The linearity values observed in the study of Tohma et al. [26] varied from 0.9901 (for GA) to 0.9995 (for vanillin and quercetin), whereas LODs and LOQs, ranged between 0.05 and 25.8 and from 0.17 to 74.5 µg/mL, respectively.

The intra- and inter-day variability for all the studied analytes was also measured to assess the precision and accuracy of the developed method. For the precision, the percent relative standard deviations (RSD %) were within the range of 1.02–6.63% and 2.48–15.00% for intra and inter-day, respectively. On the other hand, the intra-day and inter-day accuracy of various phenolic compounds varied from 80.3 to 105.7% and 89.6 to 108.1%, respectively. These results clearly indicate that high reproducibility and accuracy can be achieved by employing the aforementioned analytical method.

In Table 2, the recovery of the standard phenolic compounds added at three different matrices is shown, i.e., oregano, rosemary, and a mixed sample that consists of an equal mixture of each of the solid material remaining after the distillation of the six plants under investigation. The data revealed that recovery values were satisfactorily varying within the same range (70.0–115.0%). The lowest recovery was noticed for LUTGLU in the oregano matrix and VA for rosemary and mixed mixture. The recovery data were found to vary from 48.9–97.2% in the study of Bajkacz et al. [27], who determined 30 major active polyphenols in different plants and plant parts (lucerne, gordenrod, phacelia, buckwheat, licorice, lavender). Moreover, Baranowska and Bajkacz [28] reported recoveries of higher than 64.6% for flavonoids in dietary supplements. In general, the results obtained in the present study revealed that the developed LC-MS method was adequately precise, accurate, and reliable for the simultaneous quantitative analysis of 48 analytes present in six different aromatic plants (oregano, rosemary, sage, lemon balm, satureja, and spearmint).

### 3.2. Identification Analysis

**Figure 1:**
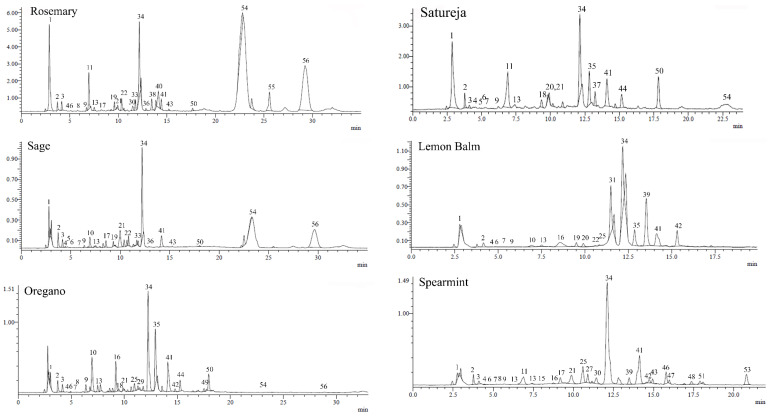
Total ion chromatogram (TIC) of the phenolic compounds identified in rosemary, oregano, sage, satureja, lemon balm, and spearmint methanol extracts by LC-ESI-DAD-MS method in negative mode. Peak numbers are as those specified in Table 3.

**Figure 2:**
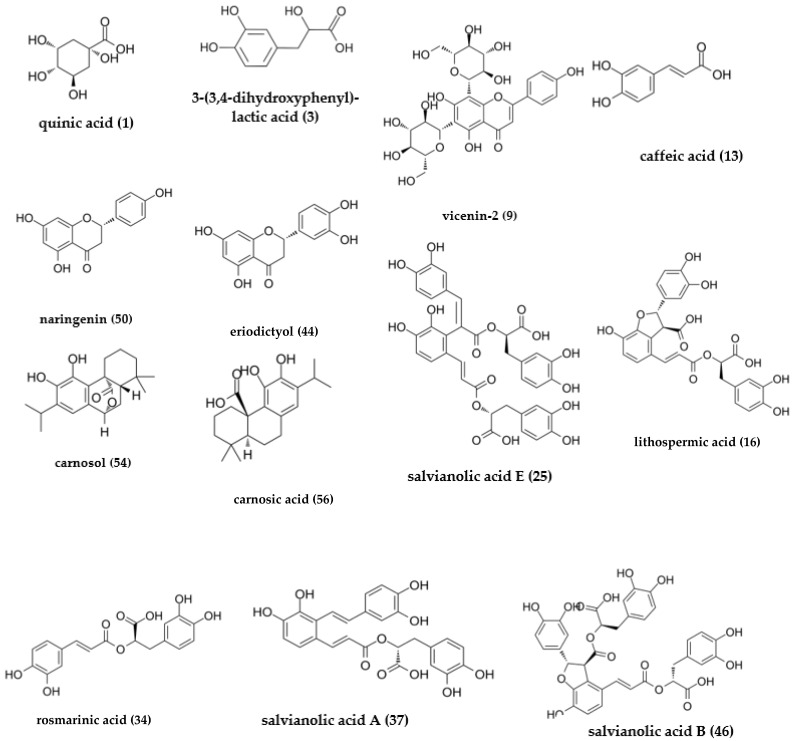
Chemical structures of the most abundant phenolic derivatives identified in the extracts of essential oil solid residues of the six plants under investigation.

Total ion chromatograms (TIC) of oregano, rosemary, sage, satureja, lemon balm, and spearmint extracts are shown in Figure 1, while the major peaks identified by LC-MS analysis are presented in Table 3. As polyphenols contain one or more hydroxyl, carboxylic acid groups, or both, MS data were acquired in the negative ionization mode. Each assignment was made after comparing retention time, UV and MS spectra data of detected compounds with reference standards or based on MS data reported in the literature and relevant databases. LC-MS analysis of the six plant extracts allowed us to identify a total of 52 different phenolic compounds (33 in rosemary, 30 in oregano, 29 in sage, 27 in satureja, 34 in lemon balm, and 35 in spearmint extracts). A total of31 of the 52 phenolic compounds were identified by comparison with reference standards. For the remaining 21 compounds, for which no standards were available, identification was based on mass measurements of the pseudomolecular [M–H]^−^ ions, the UV spectra data and by comparison with available data from the literature. Nevertheless, three compounds were not identified. Most of the compounds identified in the extracts of the six plant distillation residues were derivatives of phenolic acids, the rest were flavonoids, while two were classified as phenolic diterpenes. The chemical structures of the most abundant phenolic derivatives are presented in Figure 2. Fourteen detected compounds, such as QA, citric acid, PRCA, CLA, VIC, 4HBA, CA, APIGLU, RMA, LUT, ERY, QUE, API and NAR were found to occur in all six extracts examined. On the other hand, the phenolic diterpene ‘CARO’ ([M − H]^−^ at *m*/*z* 329), was detected in rosemary, oregano, sage, and satureja, whereas CARA ([M − H]^−^ at *m*/*z* 331), was found in rosemary, oregano, and sage.

Peak **2** (*m*/*z* 191, UV_max_ 279 nm) was characterized as citric acid, according to MS data of previous studies for similar samples [29]. Peak **3** (*m*/*z* 197, UVmax 280 nm) was tentatively identified [30,31] as dihydroxyphenyllactic acid (Danshensu) and was present in five of the six extracts (except lemon balm). This compound however, has been detected in lemon balm infusions [30] and dry spearmint as well [29,31]. Peaks **10** and **11** with precursor ion at *m*/*z* 387 and 305, respectively, in the negative mode, were tentatively characterized as medioresinol and gallocatechin isomer. Compound **10** was present in oregano and lemon balm, whereas compound **11** was detected in all extracts except oregano and sage. Medioresinol was also reported by other authors in spearmint [29] and in rosemary [32]. Peaks **16**, **35, 39** and **42** at *m*/*z* 537 with characteristic fragment ion at *m*/*z* = 493 implied the presence of lithospermic acid isomers, according to other studies [30,33].

The peaks **38** and **40** with [M-H]^−^ at *m*/*z* 503 were characterized as caffeoyl-hexosyl-hexoses, according to Celano et al. [34]. Peaks **25**, **29**, **46** and **47** with *m*/*z* 717 and peaks **37**, **51** with *m*/*z* 493 were named isomers of salvianolic acid (E, B and A, respectively) [30,31,33,34,35,36]. Peaks **31, 33** and **53** with a pseudomolecular ion [M − H]^−^ at *m*/*z* 439, 461, and 359, respectively, are tentatively identified as sulphated rosmarinic acid [30], hispidulin-7-*O*-glucoside [37], and cyclolariciresinol [31], respectively. Peak **18** occurred in oregano and satureja, peak **48** was present in spearmint and peak **55** in rosemary extracts; for these compounds, identification was not feasible.

**Table 3:** List of tentative major phenolic compounds identified by LC-MS in negative mode in rosemary (R), oregano (O), sage (S), satureja (T), lemon balm (L), and spearmint (M) distillation solid residues’ extracts, using standards or literature data as reference.

Peak	R_t_ (min)	UV_λmax_ (nm)	[Μ − H]^−^	*m/z* Main Fragments	Tentative	Reference	Extract
1	2.85	330	191	163	quinic acid	standard	R, O, S, T, L, M
2	3.72	279	191	147	citric acid	[31]	R, O, S, T, L, M
3	4.16	280	197	179	dihydroxyphenyllactic acid	[30,31]	R, O, S, T, M
4	4.33	327	353	191, 179	neochlorogenic acid	standard	R, O, S, L, M
5	4.75	270	305	191	epigallocatechin	standard	S, T
6	5.03	260, 290	153	-	protocatechuic acid	standard	R, O, S, T, L, M
7	5.42	330	353	191, 179	chlorogenic acid	standard	R, O, S, T, L, M
8	5.77	330	353	191, 179	cryptochlorogenic acid	standard	R, O, L, M
9	6.34	270, 335	593	179	vicenin-2	standard	R, O, S, T, L, M
10	6.91	285	387	305	medioresinol	[38]	O, L
11	6.96	283	305	283	gallocatechin isomer	[36]	R, T, L, M
12	7.08	260	137	-	4-hydroxybenzoic acid	standard	R, O, S, T, L, M
13	7.49	322	179	153	caffeic acid	standard	R, O, S, T, L, M
14	7.77	260	167	-	vanillic acid	standard	R, O, S, T, L
15	7.80	274	197	-	syringic acid	standard	L, M
16	9.20	253, 344	537	493	lithospermic acid	[30]	O, L, M
17	9.23	260, 360	593	287	luteolin-7-O-rutinoside	standard	R, S, T, M
18	9.35	266, 337	431	329	n.i.		O, T
19	9.58	274	597	493, 345, 179	yunnaneic acid F	[30]	R, S, L
20	9.90	253, 366	447	285, 329	luteolin-7-O-glucoside	standard	R, S, T, L
21	9.94	254, 346	461	285	luteolin-7-O-glucuronide	[2,30,35,37,39]	R, O, S, T, M
22	10.33	272,345	477	289	isorhamnetin-3-O-D-glucoside	standard	R, S, L
23	10.35	280	623	-	verbascoside	standard	R, O, S, T, L
24	10.41	327	515	353, 179	3,4-dicaffeoylquinic acid	standard	L, M
25	10.64	283,345	717	519	salvianolic acid E	[30,34,35]	O, L, M
26	10.65	323	193	163	ferulic acid	standard	L, M
27	10.90	327	515	353, 179	3,5-dicaffeoylquinic acid	standard	M
28	11.39	260, 360	431	359, 193	apigenin-7-O-glucoside	standard	R, O, S, T, L, M
29	11.49	289, 330	717	431	salvianolic acid E isomer	[34,36]	O, M
30	11.50	283	609	301	hesperidin	standard	R, M
31	11.51	326	439	285, 403, 345	sulphated rosmarinic acid	[30]	L
32	11.60	327	515	353, 179	4,5-dicaffeoylquinic acid	standard	L, M
33	11.76	332	461	283	hispidulin-7-O-glucoside	[37]	R, S
34	12.15	330	359	197	rosmarinic acid	standard	R, O, S, T, L, M
35	12.72	321	537	493	lithospermic acid isomer	[33]	O, T, L, M
36	13.00	291	287	169	dihydrokaempferol	standard	R, O, S, T
37	13.24	286, 329	493	359	salvianolic acid A	[30,33]	L
38	13.44	269, 337	503	285	caffeoyl-hexosyl-hexose	[34]	R
39	13.53	293, 326	537	493, 359	lithospermic acid isomer	[30]	L
40	13.86	269, 337	503	285	caffeoyl-hexosyl-hexose	[34]	R
41	14.12	240	137	-	salicylic acid (IS)	standard	IS
42	14.79	287, 325	537	493, 359	lithospermic acid isomer	[33]	O, T, M
43	15.15	282	285	-	luteolin	standard	R, O, S, T, L, M
44	15.20	287	287	269, 169	eriodictyol	standard	R, O, S, T, L, M
45	15.38	256, 370	301	285	quercetin	standard	R, O, S, T, L, M
46	15.79	286, 322	717	519	salvianolic acid B	[14]	M
47	15.99	286, 322	717	519	salvianolic acid B isomer	[31]	M
48	17.37	294, 333	329	283	n.i.		M
49	17.63	268, 337	269	191	apigenin	standard	R, O, S, T, L, M
50	17.83	288	271	253, 193, 153	naringenin	standard	R, O, S, T, L, M
51	17.91	274, 328	493	271	salvianolic acid A isomer	[31]	M
52	18.26	264, 360	285	169	kaempferol	standard	R, O, S, T, L
53	20.80	278, 346	359	329	cyclolariciresinol	[31]	M
54	22.80	280	329	285	carnosol	standard	R, O, S, T
55	25.57	266, 340	283	269	n.i.		R
56	29.07	280	331	287	carnosic acid	standard	R, O, S

n.i.—not identified; IS—internal standard.

### 3.3. Quantitative Analysis

Validation was performed by applying the developed LC-MS method for the simultaneous determination of 48 targeted analytes including 20 phenolic acids, 26 flavonoids, and 2 phenolic diterpenes in the methanolic extracts of plant residues from six different aromatic plants of the *Lamiaceae* family obtained after removal of the essential oils as reported in Section 2.3 and Section 2.4, respectively.

The results from quantitative measurements are presented in Table 4. The content in phenolic compounds in the six samples decreased in the following range: spearmint > oregano > lemon balm = rosemary > sage > satureja. Not all the 48 compounds were detected and quantified in the extracts of the six distillation solid residues of the aromatic plants, whereas the concentrations of the analytes studied varied among the solid residues of the six plants. In general, the percentage of phenolic acids in the total content of phenolic compounds detected was higher than that of flavonoids and phenolic diterpenes, except for the rosemary solid residues, where the content of phenolic diterpenes surpassed that of phenolic acids. Among the studied extracts, the highest amount of phenolic acids appeared in spearmint followed by lemon balm, oregano, sage, and finally satureja and rosemary, between which no statistical difference was found. From the group of total diterpenes, in spearmint and lemon balm, both CARO and CARA are missing, whereas CARA was not detected in Satureja. On the other hand, rosemary had the highest amount of phenolic diterpenes (6175.2 ± 11.7 mg/100 g) among the plant solid residues studied. The results showed that satureja contains the highest amount of total flavonoids (3112.9 ± 90.9 mg/100 g) among all six samples examined, whereas sage has the lowest (747.6 ± 13.6 mg/100 g).

Among the derivatives of phenolic acids, RMA was the dominant compound in the six samples, ranging from 2469.6 mg/100 g in rosemary to 9660.0 mg/100 g in spearmint, with the latter containing, in addition, a total of 3891.9 mg/100 g of RMA derivatives (Sulphated RMA, LITHI and LITHII). Similarly, oregano, satureja, and lemon balm contained a high amount of RMA derivatives (4033.5, 585.2, and 1415.7 mg/100 g, respectively).

In their study, Celano et al. [34] reported that residual wastewaters obtained after essential oil distillation of rosemary and sage, contained large amounts of RMA (46.8 ± 9.4 mg/100 mL and 135.3 ± 12.3 mg/100 mL, respectively) and other phenolic compounds (mainly caffeic acid derivatives and flavonoid glycosides). The amount of RMA present in the solid extract of the plants after essential oil extraction is dependent on the degree of partial degradation of RMA, due to the high process temperatures applied, as well as the solubilization in distillation water and its subsequent removal with the waste water stream. The same conclusion could be drawn in general for the phenolics, suggesting the importance of applying a multistep biorefining scheme for recovery of these valuable compounds.

The second highest in concentration compound within the group of phenolic acids derivatives, present in all the six plant residues, was QA, showing the highest level in rosemary (1056.4 ± 26.8 mg/100 g) and the lowest in lemon balm (262.0 ± 22.0 mg/100 g). CLA was detected in all samples, but it was not quantified in satureja, whereas the spearmint appeared to have the highest CLA content, followed by oregano, sage, and rosemary. Similarly, spearmint extracts had the greatest contents of nCLA and cCLA, followed by rosemary and oregano, whereas cCLA was not detected in sage, satureja, and lemon balm. Most of the extracts studied were also found to contain CA and 4HBA in significant amounts. On the other hand, FA was present only in spearmint and lemon balm, with the former showing a relatively high amount of 152.5 ± 2.5 mg/100 g.

Total flavonoids content decreased in the following order: satureja > oregano > spearmint > rosemary > lemon balm > sage. Flavonoid compounds detected in the six extracts varied largely in their amount among the extracts. Derivatives of GCAT were the most abundant compounds in all extracts, except for sage, where they were not detected. The GCAT isomer was found in rosemary (2111.9 ± 97.5 mg/100 g), satureja (2805.8 ± 74.2 mg/100 g), and spearmint (2332.8 ± 63.2 mg/100 g), representing 81.2%, 90.1% and 82.2% of the total flavonoids in the respective extracts. On the other hand, medioresinol, the other derivative of GCAT, was the most abundant flavonoid in oregano (1708.4 ± 110.4 mg/100g) and lemon balm (800.0 ± 20.0 mg/100 g) reaching 57.5% and 71.2% of the total flavonoids, respectively. In contrast, sage which contained the lowest amount of total flavonoids among the studied extracts, showed a more even distribution in the amount among different flavonoids. Flavonoid compounds such as VIC (252.0 ± 11.7 mg/100 g), LUTGLU (207.2 ± 3.2 mg/100 g) and LUTRUT (132.6 ± 1.4 mg/100 g) represented 79.2% of the total flavonoid present in the sage extract (33.7%, 27.7% and 17.7%, respectively). LUTRUT was also present in rosemary (199.7 ± 9.3 mg/100 g) and spearmint (284.4 ± 8.4 mg/100 g), whereas LUTGLU was found only in lemon balm (186.6 ± 2.2 mg/100 g). VIC was present in high concentrations in oregano (888.4 ± 24.4 mg/100 g). From the total of 48 phenolics detected by the developed analytical method, none of the following compounds were detected in the six studied extracts: GA, GNA, SRA, pCA, SA, can, EPI, CAT, NARI, MYR, CRY, and GAL.

### 3.4. Phenolic and Flavonoid Content—Antioxidant Activity

**Figure 3:**
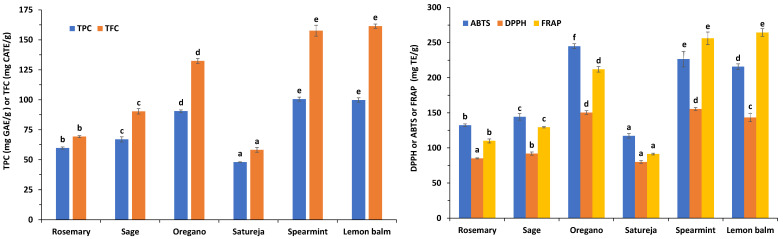
Total phenolics content (TPC), total flavonoids content (TFC) and antioxidant activity as evaluated by DPPH, ABTS and FRAP tests in oregano, rosemary, sage, satureja, lemon balm, and spearmint methanol extracts of the plant residues, following extraction of the respective essential oil. Different superscripts letters above the error bars for each reported parameter indicate significant differences (*p* ≤ 0.05) among the means, as determined by the Duncan’s multiple range test.

The amount of total phenolics content (TPC) as well as total flavonoids content (TFC) in the six extracts was determined spectrophotometrically by the Folin-Ciocalteu’s phenol reagent method and aluminum complexation assay, respectively. Figure 3 reveals that the TPC and TFC values in lemon balm were similar to that of spearmint and decreased significantly in the following order: lemon balm = spearmint > oregano > sage > rosemary > satureja. The TPC and TFC values ranged from 47.91 to 100.53 mg GAE/g and 58.17 to 161.42 mg CATE/g, respectively. TPC values of spearmint (100.53 mg GAE/g), lemon balm (99.76 mg GAE/g), oregano (90.49 mg GAE/g), sage (66.92 mg GAE/g), rosemary (59.74 mg GAE/g), and satureja (47.91 mg GAE/g) recorded in the present study are in agreement with the data available in scientific literature [9,10,33,34]. The same trend was also noted for the TFC values.

Determination of antioxidant potential of plant extracts is of great importance for the estimation of the protective role their components can be play in foods and biological systems. The antioxidant capacities of extracts were evaluated by DPPH, FRAP, and ABTS assays and the results are also presented in Figure 3. It was noted that the hydrogen-donating ability of the spearmint extract to oxidize DPPH was similar to that of oregano, and significantly higher than the other extracts. Specifically, the antioxidant activity of samples, based on the DPPH assay, decreased significantly in the following order: spearmint > oregano > lemon balm > sage > rosemary > satureja. A similar trend was observed for the data derived by the ABTS test. Oregano exhibited the highest hydrogen-donating ability against ABTS+ radical followed by spearmint and lemon balm, sage, rosemary, and satureja. Lemon balm and spearmint showed the highest ability to donate electrons and reduce Fe^3+^ to Fe^2+^, thus increasing FRAP values followed by oregano, sage, rosemary, and finally satureja.

### 3.5. Correlation Analysis

TPC and TFC correlated very well with antioxidant activity of extracts, as determined by the ABTS, DPPH and FRAP assays, showing very high positive correlation coefficients (0.939, 0.961, and 0.984 for TPC, and 0.919, 0.952, and 0.994 for TFC, respectively, at *p* ≤ 0.01 level of significance (2-tailed)) (Table 5). These correlation values are similar to those previously reported by Skendi et al. [1]. TPC correlated positively (0.795 at *p* ≤ 0.01, (2-tailed)) with values of total phenolics obtained by LC-MS. In contrast, the TFC does not show a significant correlation with the total flavonoids obtained from LC-MS. Instead, it correlated negatively with diterpenes and positively with total phenolics measured by LC-MS (−0.611 and 0.736, respectively at *p* ≤ 0.01, (2-tailed)). It was also observed that total phenolics obtained from LC-MS showed a slightly weaker correlation with ABTS, DPPH, and FRAP compared to the total phenolic acids (0.809 vs. 0.925; 0.823 vs. 0.974; 0.745 vs. 0.956 *p* ≤ 0.01, respectively). In general, the antioxidant assays showed no correlation with total flavonoids obtained by the LC-MS method, whereas the phenolic diterpenes showed significant but much lower negative correlation coefficients (−0.563, −0.633, and −0.629 for ABTS, DPPH, and FRAP, respectively). In our previous study, we have noted no correlation between the antioxidant activity measured by ABTS, DPPH, and FRAP assays and the total phenolics content as determined by HPLC analysis [1]. This could be due to the lower number of analytes quantified (24 vs. 48) with the abovementioned method compared to the present work.

It seems that is mainly the group of phenolic acid derivatives that contributes positively to increase in the antioxidant activity evaluated by the ABTS and DPPH assays. On the other hand, the FRAP assay could be linked with the presence of flavonoids in the dried residues of the aromatic plants of *Lamiaceae* family, following the essential oil extraction.

## 4. Conclusions

In the present study, we developed and validated an analytical method for the detection and quantification of 48 phenolic compounds in the dry residues obtained after steam distillation of six common aromatic plants of the Lamiaceae family (oregano, rosemary, sage, satureja, lemon balm, and spearmint). The applied extraction procedure for obtaining the phenolics from the dried solid residuals after steam distillation is simple and efficient, followed by a sensitive and accurate LC-MS method for the qualitative and quantitative determination. In general, a total of 55 different compounds were detected in the extracts of the six essential oils’ solid residues and 52 of these were identified. Application of the method on residues of the aforementioned plants revealed the presence and allowed the quantification a total of 38 compounds, including 17 phenolic acids, 19 flavonoids, and 2 phenolic diterpenes. The total amount of phenolics decreased in the following order: spearmint, oregano, rosemary, lemon balm, sage, satureja. Correlation analysis showed that a higher contents of phenolics indicated higher antioxidant activity. The research findings also suggest that the variation in antioxidant activity of the extracts was mostly due to the presence of phenolic acid derivatives, rather than flavonoids. Among the three assays employed to assess antioxidant activity, the ABTS, DPPH, and FRAP mainly reflect the content of phenolic acid derivatives and total phenolics present in the solid residues, whereas no correlation was noted with total flavonoid content.

Overall, the results of our study disclosed that waste residues of the essential oil industry would be a potential source of valuable polyphenolic compounds with potential applications in several industrial sectors, such as food, cosmetics, and pharmaceuticals, as bioactive ingredients in different formulations. The valorization of distillation wastes, through the recovery of valuable phytochemicals, will assist sustainable development, providing environmental benefits and promoting the bioeconomy.

## Figures and Tables

**Table 1:** Main validation data for the targeted phenolic compounds determined by LC-MS method.

Analytes	Rt (min)	[Μ − H]^−^ (*m*/*z*)	UV_max_ (nm)	Equation	R^2^	Linearity Range (ng/mL)	LOD (ng/mL)	LOQ (ng/mL)	Precision (RSD%)		Accuracy (%)	
									Intra-Day	Inter-Day	Intra-Day	Inter-Day
QA	2.85	191	330	y = 0.2493 x + 0.0018	0.9981	10–4000	21.6	65.4	2.14	6.10	102.2	102.3
GA	3.80	169	272	y = 0.1508x + 0.0002	0.9979	10–4000	25.0	75.7	3.10	6.81	100.2	101.5
1-CQA	4.00	353	327	y = 0.0412x − 0.0016	0.9987	10–4000	8.9	27.0	3.05	6.52	89.5	95.3
GCAT	4.08	305	270	y = 0.0437x − 0.0021	0.9985	10–4000	21.1	64.2	3.17	13.41	104.1	105.2
nCLA	4.33	353	325	y = 0.0614x − 0.0003	0.9995	10–4000	29.4	89.2	2.91	3.91	97.4	96.8
EGCAT	4.75	305	270	y = 0.0355x − 0.0012	0.9978	10–4000	29.7	90.0	2.18	9.47	103.2	105.0
PRCA	5.03	153	260	y = 0.2319x + 0.0241	0.9990	10–10,000	51.1	155.1	2.56	5.41	94.9	94.3
CLA	5.42	353	330	y = 0.0848x + 0.0047	0.9999	50–10,000	18.6	56.3	3.02	3.85	100.6	101.5
cCLA	5.77	353	330	y = 0.0444x + 0.0069	0.9993	50–10,000	27.9	84.5	3.06	8.82	99.2	101.0
CAT	5.95	289	280	y = 0.0588x + 0.0011	0.9957	10–4000	25.2	76.4	2.29	5.26	101.7	102.7
VIC	6.34	593	270, 335	y = 0.0537x − 0.0032	0.9981	50–4000	33.4	101.2	1.45	7.57	100.5	100.7
4HBA	7.08	137	260	y = 0.0109x + 0.0157	0.9953	500–50,000	164.8	499.5	3.02	7.80	100.6	104.5
GNA	7.10	153	327	y = 0.3942x + 0.0122	0.9997	50–4000	54.0	163.7	3.25	3.42	101.8	100.5
EPI	7.24	289	280	y = 0.0714x + 0.0064	0.9993	50–5000	50.7	153.8	5.36	9.07	101.2	102.1
CA	7.49	179	322	y = 0.3171x + 0.0789	0.9977	10–10,000	51.6	156.4	2.08	5.47	102.6	104.4
VA	7.77	167	260	y = 0.0033x − 0.0071	0.9985	500–50,000	164.3	497.9	5.60	10.47	95.3	90.3
SRA	7.80	197	274	y = 0.0017x − 0.0024	0.9981	500–50,000	74.9	227.0	5.59	11.71	101.3	94.8
RUT	9.11	609	256, 354	y = 0.1427x + 0.0087	0.9989	10–4000	30.6	92.7	2.43	3.32	100.6	101.5
LUTRUT	9.23	593	260, 360	y = 0.1678x + 0.0098	0.9961	10–4000	10.0	30.3	3.77	4.81	99.6	102.5
QUEGLU	9.63	463	260, 360	y = 0.1408x + 0.0037	0.9995	10–4000	92.9	281.6	2.97	3.34	97.1	96.1
HYP	9.77	463	260, 360	y = 0.0487x + 0.0032	0.9969	10–4000	17.8	54.0	6.07	8.61	101.0	103.1
pCA	9.80	163	309	y = 0.1153x − 0.0469	0.9941	50–10,000	168.5	510.7	3.70	9.81	95.2	96.7
LUTGLU	9.87	447	253, 366	y = 0.0570x + 0.0032	0.9962	50–5000	25.8	78.3	4.89	7.57	101.2	102.0
ISRUT	10.33	623	254, 353	y = 0.1333x − 0.0015	0.9960	50–4000	61.7	187.1	2.26	4.85	105.6	108.1
VER	10.35	623	280	y = 0.0382x − 0.0029	0.9921	10–4000	57.5	174.3	1.46	7.66	93.2	95.9
3,4-DCQA	10.41	515	327	y = 0.2283x − 0.0022	0.9999	50–5000	36.1	109.3	4.38	7.68	104.3	108.0
FA	10.65	193	323	y = 0.0024x − 0.0004	0.9994	1000–40,000	208.1	630.7	4.65	11.92	96.0	99.5
3,5-DCQA	10.90	515	327	y = 0.1035x − 0.0362	0.9990	50–4000	9.7	29.4	3.61	6.51	89.2	92.6
ISGLU	11.01	477	254, 333	y = 0.5449x − 0.0558	0.9981	50–4000	15.1	45.6	5.57	8.52	90.3	95.3
SA	11.04	223	323	y = 0.0051x + 0.0069	0.9951	1000–40,000	132.2	400.7	3.57	10.61	97.1	99.3
NARI	11.10	579	284	y = 0.1434x − 0.0017	0.9960	10–4000	64.5	195.5	2.04	7.73	100.6	99.7
APIGLU	11.39	431	260, 360	y = 0.2912x + 0.0125	0.9984	10–4000	49.0	148.7	1.45	7.73	99.9	99.6
HESP	11.50	609	283	y = 0.2518x + 0.0249	0.9995	500–4000	61.2	185.4	4.02	9.02	91.6	95.0
4,5-DCQA	11.60	515	327	y = 0.1923x − 0.0066	0.9996	500–4000	6.9	20.8	2.01	5.32	80.3	89.6
RMA	12.15	359	330	y = 0.1179x + 0.0133	0.9987	10–10,000	16.4	49.8	2.92	4.58	104.9	105.7
MYR	12.53	317	253, 372	y = 0.4649x + 0.0459	0.9963	10–5000	20.2	61.4	4.76	9.54	99.3	106.1
DHKAE	12.88	287	291	y = 0.9467x + 0.0267	0.9986	10–4000	10.7	32.4	5.63	8.65	100.5	106.3
ERY	15.15	287	287	y = 0.3298x + 0.0247	0.9935	10–4000	6.1	18.5	2.88	7.52	98.9	100.2
LUT	15.17	285	253, 366	y = 0.7956x + 0.0273	0.9961	10–5000	7.7	23.4	2.95	2.48	99.1	99.2
QUE	15.38	301	256, 370	y = 0.7646x + 0.0053	0.9962	10–2000	12.0	36.3	1.02	3.81	92.1	94.1
CNA	17.11	147	276	y = 0.0007x + 0.0037	0.9966	1000–40,000	24.1	72.9	6.11	7.45	101.5	93.6
API	17.63	269	268, 337	y = 1.0850x + 0.0619	0.9978	10–4000	33.9	102.3	3.11	3.81	98.1	94.6
NAR	17.83	271	288	y = 0.9427x − 0.0209	0.9987	10–4000	45.6	138.3	5.11	5.95	101.6	97.4
KAE	18.26	285	264, 360	y = 1.3159x + 0.2258	0.9956	10–5000	168.4	510.3	1.43	3.81	100.9	103.0
CARO	22.50	329	280	y = 0.3152x − 0.5500	0.9974	50,000–200,000	421.9	1278.7	2.20	12.11	93.0	93.8
CRY	24.90	253	287	y = 1.0394x + 0.0080	0.9944	10–4000	29.9	90.8	4.53	5.91	97.8	104.4
GAL	26.00	272	265, 358	y = 1.1780x + 0.1237	0.9953	10–5000	80.9	245.2	3.38	4.46	104.9	100.5
CARA	29.07	331	280	y = 0.1733x + 0.3744	0.9992	25,000–200,000	718.3	2176.7	6.63	15.00	105.7	106.7

**Table 2:** Recovery results of 48 phenolic compounds in oregano, rosemary and a mixture sample (equal quantities of oregano, rosemary, sage, satureja, lemon balm, and spearmint) applying the developed LC-MS method.

Analytes	Recovery (%)			Analytes	Recovery (%)		
	Oregano	Rosemary	Mixed		Oregano	Rosemary	Mixed
QA	101.9	96.0	85.0	CAT	115.0	108.2	112.7
GA	72.0	75.3	73.6	EPI	105.0	107.3	109.4
1-CQA	92.4	76.5	91.5	GCAT	80.0	81.3	76.5
nCLA	89.4	76.9	83.5	EGCAT	91.2	75.4	86.4
PRCA	88.3	84.0	87.2	VIC	99.5	81.8	94.5
CLA	78.3	80.0	86.4	RUT	88.7	87.4	92.0
cCLA	101.6	107.0	110.0	LUTRUT	82.3	70.5	85.3
VA	75.5	70.2	70.06	LUTGLU	70.0	71.3	78.0
4HBA	84.2	88.2	90.6	QUECLU	83.3	86.2	87.0
SRA	70.5	77.0	71.3	HYP	99.2	78.0	99.0
CA	96.2	90.6	103.0	VER	90.9	97.5	99.3
pCA	98.8	91.5	98.3	ISRUT	104.5	115.0	109.5
FA	108.0	102.3	117.2	ISGLU	82.5	72.6	70.3
SA	70.4	71.3	70.6	NARI	74.2	72.7	76.5
3,4-DCQA	110.3	105.3	101.9	APIGLU	71.8	83.2	70.6
3,5-DCQA	108.0	104.0	110.0	HESP	83.3	87.1	76.7
4,5-DCQA	107.1	107.3	102.2	MYR	86.3	90.0	83.3
RMA	114.6	109.0	107.8	DHKAE	71.3	75.7	70.6
GNA	79.0	82.3	84.6	ERY	99.4	80.5	100.0
CNA	82.5	70.5	71.3	LUT	82.8	77.9	89.0
CRY	110.0	88.8	70.6	QUE	90.4	80.1	85.0
GAL	89.7	80.8	86.9	API	99.8	93.2	84.4
CARO	80.6	70.5	72.2	KAE	95.6	90.6	92.6
CARA	85.3	113.2	98.0	NAR	85.3	107.2	109.3

**Table 4:** Major phenolic compounds identified and quantified, with the targeted method, in the solid residues, following steam distillation of the respective aromatic plants, expressed as mg/100g.

Analytes	Rosemary	Sage	Oregano	Satureja	Spearmint	Lemon Balm
QA	1056.4 ± 26.8	288.3 ± 1.6	579.0 ± 17.0	698.0 ± 38.8	419.8 ± 38.2	262.0 ± 22.0
1-CQA	50.0 ± 4.0	nd	5.4 ± 0.3	nd	14.9 ± 0.6	nd ± 0.0
nCLA	33.1 ± 2.4	7.6 ± 0.0	20.1 ± 0.6	7.1 ± 0.3	81.7 ± 1.1	8.8 ± 0.0
PRCA	51.1 ± 3.1	54.8 ± 0.8	100.0 ± 2.0	17.4 ± 0.2	39.8 ± 0.3	25.6 ± 0.6
CLA	0.6 ± 0.1	7.2 ± 0.5	9.5 ± 0.0	<LOQ	19.4 ± 1.4	0.2 ± 0.1
cCLA	46.8 ± 2.0	nd	2.6 ± 0.1	nd	75.9 ± 2.1	<LOQ ± 0.0
VA	38.9 ± 1.4	20.8 ± 0.2	34.6 ± 0.6	24.7 ± 0.4	nd	26.2 ± 0.9
4HBA	34.1 ± 1.1	29.7 ± 0.5	44.8 ± 0.5	26.3 ± 1.0	25.5 ± 0.9	25.5 ± 0.9
CA	37.4 ± 2.6	14.1 ± 0.3	16.6 ± 0.4	11.7 ± 1.3	36.2 ± 0.5	36.4 ± 1.3
3,4-DCQA	nd	nd	nd	nd	2.7 ± 0.1	1.8 ± 0.1
3,5-DCQA	nd	nd	nd	nd	31.9 ± 0.1	nd ± 0.0
4,5-DCQA	nd	nd	nd	nd	10.0 ± 0.0	21.0 ± 1.0
FA	nd	nd	nd	nd	152.5 ± 2.5	21.0 ± 1.0
RMA	2469.6 ± 48.8	4251.6 ± 160.4	5914.8 ± 253.2	2530.0 ± 30.0	9660.0 ± 60.0	9330.4 ± 147.2
Sulphated RMA	nd	nd	nd	nd	3444.5 ± 0.0	nd ± 0.0
LITA isomer I	nd	nd	1366.7	nd	150.7 ± 9.0	640.0 ± 16.0
LITA isomer II	nd	nd	2666.8 ± 38.3	585.2 ± 14.8	296.7 ± 15.3	775.7 ± 15.9
Phenolic acids	3817.9 ^a^ ± 92.0	4674.1 ^b^ ± 159.1	10760.9 ^c^ ± 142.3	3900.3 ^a^ ± 25.1	14462.2 ^e^ ± 34.9	11174.6 ^d^ ± 140.3
EGCAT	nd	46.0 ± 2.0	nd	<LOQ	nd	nd
medioresinol	nd	nd	1708.4 ± 110.4	nd	nd	800.0 ± 20.0
GCAT isomer	2111.9 ± 97.5	nd	nd	2805.8 ± 74.2	2332.8 ± 63.2	96.3 ± 2.7
VIC	28.7 ± 0.9	252.0 ± 11.7	888.4 ± 24.4	79.5 ± 1.5	18.6 ± 0.3	6.8 ± 0.1
LUTRUT	199.7 ± 9.3	132.6 ± 1.4	nd	nd	284.4 ± 8.4	nd
LUTGLU	nd	207.2 ± 3.2	nd	nd	nd	186.6 ± 2.2
VER	70.8 ± 10.8	10.6 ± 0.6	<LOQ	19.7 ± 0.3	12.0 ± 0.6	<LOQ
HYP	nd	nd	<LOQ	nd	nd	17.6 ± 0.8
QUEGLU	nd	nd	<LOQ	5.4 ± 0.2	<LOQ	nd
DHKAE	5.5 ± 0.3	<LOQ	29.8 ± 1.5	5.9 ± 0.9	nd	nd
ISRUT	nd	38.4 ± 1.6	nd	nd	56.9 ± 0.7	nd
ISGLU	114.0 ± 4.1	37.2 ± 1.2	nd	<LOQ	<LOQ	<LOQ
APIGLU	5.0 ± 0.4	15.7 ± 0.2	1.6 ± 0.0	<LOQ	<LOQ	17.4 ± 0.6
ERY	nd	nd	185.2 ± 7.8	98.2 ± 15.6	<LOQ	<LOQ
LUT	1.3 ± 0.1	3.5 ± 0.2	2.0 ± 0.3	0.2 ± 0.0	3.6 ± 0.2	1.4 ± 0.1
QUE	<LOQ	<LOQ	2.9 ± 0.4	<LOQ	<LOQ	<LOQ
HESP	58.6 ± 3.4	nd ± 0.0	nd	nd	124.2 ± 7.8	nd
API	5.8 ± 0.5	4.5 ± 0.4	16.8 ± 0.1	4.0 ± 0.2	0.1 ± 0.0	<LOQ
NAR	<LOQ	<LOQ	140.8 ± 3.2	94.3 ± 1.3	5.6 ± 0.2	<LOQ
Flavonoids	2601.3 ^c^ ± 103.9	747.6 ^a^ ± 13.6	2976.0 ^e^ ± 73.5	3112.9 ^f^ ± 90.9	2838.1 ^d^ ± 46.4	1126.0 ^b^ ± 21.9
CARO	2211.6 ± 228.4	2501.2 ± 199.6	485.6 ± 10.4	544.0 ± 16.0	nd	nd
CARA	3963.6 ± 240.1	1631.2 ± 35.4	737.6 ± 24.0	nd	nd	nd
Phenolic diterpenes	6175.2 ^d^ ± 11.7	4132.4 ^c^ ± 164.2	1223.2 ^b^ ± 13.6	544.0 ^a^ ± 16.0	0.0	0.0
Total	12594.3 ^c^ ± 207.5	9554.2 ^b^ ± 336.9	14960.2 ^d^ ± 55.2	7557.2 ^a^ ± 100.0	17300.3 ^e^ ± 77.9	12300.5 ^c^ ± 118.3

nd—not detected; LITA—lithospermic acid; <LOQ—lower than the level of quantification; different superscripts letters in the same line indicate differences (*p* ≤ 0.05) amongst the means, as determined by the Duncan’s multiple range test.

**Table 5:** Pearson’s correlations between polyphenolic contents of the studied extracts and their different corresponded antioxidant activities.

Variables	TFC	ABTS	DPPH	FRAP	Phenolic Acids	Diterpenes	LC-MS Phenolics
TPC	0.992 **	0.939 **	0.961 **	0.984 **	0.949 **	−0.539 *	0.795 **
TFC		0.919 **	0.952 **	0.994 **	0.950 **	−0.611 **	0.736 **
ABTS			0.981 **	0.916 **	0.925 **	−0.563 *	0.809 **
DPPH				0.956 **	0.974 **	−0.633 **	0.823 **
FRAP					0.956 **	−0.629 **	0.745 **

* Correlation is significant at the 0.05 level (2-tailed); ** Correlation is significant at the 0.01 level (2-tailed).

## Data Availability

Data is contained within the article.
